# Language Concordance and Interpreter Use for Spanish-Preferring Patients: Qualitative Study of Perspectives from Primary Care Providers

**DOI:** 10.1007/s11606-025-09414-9

**Published:** 2025-02-10

**Authors:** Denise D. Quigley, Nabeel Qureshi, Efrain Talamantes, Zachary Predmore

**Affiliations:** 1 RAND Corporation, Santa Monica, CA USA; 2 AltaMed Health Services Corporation, Los Angeles, CA USA; 3 RAND, Boston, MA USA

**Keywords:** patient experience, primary care providers, quality of care, spanish-preferring patients, language concordance, interpreter use

## Abstract

**Background:**

Poor quality communication and language barriers lead to worse care experiences and inferior health care outcomes for those with limited English proficiency. Fewer than one-third of outpatient providers regularly use professional interpreters when communicating with non-English preferring patients. Effective strategies to address language barriers in primary care are lacking and in demand.

**Objective:**

Examine provider perspectives on providing in-person care to Spanish-preferring patients.

**Design:**

Partnered with a large, urban Federally Qualified Health Center predominantly caring for Spanish-preferring Hispanic patients, we identified primary care providers who (1) were language-concordant (provider and patient speak same language); (2) used qualified interpreters; and (3) used informal strategies for interpretation/communication.

**Participants:**

We interviewed 24 providers (10 language-concordant, 9 who used qualified interpreters, 5 who used informal interpreters and other strategies; response-rate 23%).

**Approach:**

We established codes using systematic, inductive procedures to generate insights from responses and identified themes using content analysis.

**Results:**

Providers—both language-concordant and those using interpreters—preferred to speak the same language as the patient, employed varying communication strategies, and required more time to care for Spanish-preferring patients for differing reasons. Using interpreters did not always improve communication because using qualified interpreters requires more time for initiating interpretation, connectivity issues, and conducting consecutive interpretation; using any interpreter requires provider-interpreter clarification or staff to translate, and sometimes interpreters had difficulty with medical content/terminology. Provider-patient visits also qualitatively differed based on language spoken and interpreter use in eliciting concerns, topics covered, patient comprehension, and time spent on rapport-building and patient education.

**Conclusions:**

Providers described barriers that organizations need to address to facilitate effective communication and language interpretation when caring for Spanish-preferring patients. Research is needed that identifies and tests language support strategies for providers and clinics and structural changes that preserve time during patient visits for providers and patients to spend on health care needs.

## INTRODUCTION

Medical interpreters can bridge communication gaps. Health care organizations that receive federal funding are legally required to provide qualified professional interpreter services to individuals with limited English proficiency.^1^ Many other patients besides those with limited English proficiency may prefer to speak in a non-English language during a medical encounter. Still, non-English-preferring patients are not always ensured access and support from interpreters. Nationally, fewer than one-third of outpatient physicians reported regularly using trained professional interpreters when communicating with non-English-preferring patients.^2^ This poses challenges to providing high-quality communication for non-English-preferring patients.

Effective communication is a critical aspect of delivering high-quality care.^3–10^ Poor-quality communication with providers and nurses leads to poor clinical experiences and inferior health care outcomes.^11–14^ Language barriers, inadequate interpreter use, and poor provider-patient communication are associated with more serious medical events such as prescription drug complications.^15^ Ample research has shown that language access services, whether via language-concordant clinicians or professional interpreters, are crucial to ensure more equitable care and outcomes for non-English-preferring patients;^16,17^ however, the evidence in primary care is limited and also less robust for examining patient experience related to language concordance and interpreter use.^18–20^

Strategies to support effective provider-patient communication for non-English-proficient patients in primary care are suboptimal. Providing on-site professional interpreters in busy clinics may be expensive and delay care.^21,22^ Also, interpreters are not reimbursed by most private insurances outside of California and only minimally reimbursed by Medicaid in 13 states.^23^ Using family and friends as interpreters can compromise patient confidentiality and result in misinterpretation of medical terms (in addition to being illegal). Using bilingual employees takes these staff away from their work, impacting the flow and pace of providing care. Patients who need but do not get interpreters have poorer understanding of their diagnosis and treatment plan and frequently wish their provider had explained things better.^16^ Language-concordant providers, i.e., those that speak the same language as the patient, are preferred and are perceived to provide the best medical care and care experience,^17,24,25^ but the pool of providers who are qualified as bilingual is inadequate.^26^

As of 2020, 19% (62.1 million) of the United States (U.S.) population was Hispanic, increasing from 16% (50.5 million) in 2010.^27^ The Hispanic population is estimated to reach 106 million, consisting of roughly 30% of the U.S. population, by 2050.^28^ In addition, the 2020 U.S. census estimated that 28% of Hispanics in the U.S. have limited English proficiency (29.7 million). These demographic shifts have consequences for high-quality health care delivery. As the Hispanic population grows, with a significant portion preferring Spanish in part due to limited English proficiency, health care organizations will continue to be faced with the challenges of ensuring high quality patient-provider communication and determining whether they are providing high-quality language services to their patients and families.

Effective strategies to support the language needs of the growing Hispanic, Spanish-preferring patient population are needed as health status and access to health care may be compromised by difficulty in communicating medical needs to providers who do not speak a patient’s preferred language.^11,16,29,30^ Research to supply health care organizations with effective language support strategies for primary care is lacking and increasingly in demand across the U.S.

A systematic review of evidence focused on primary care experiences of Spanish-preferring patients^31^ found little evidence on effective strategies or interventions. The evidence that does exist showed that provider-patient language concordance, i.e., patient and provider speak the same language, is associated with better patient experience. Using high-quality interpreters may improve patient experience. Still, patients with Spanish-language preference indicated worse experiences (e.g., long waits, problems getting appointments, and not understanding nurses). Altogether, the review highlighted the need for strategies to eliminate disparities and enhance communication for all Spanish-preferring primary care patients. In this work, we examined perspectives of primary care providers caring for Spanish-preferring patients.

## METHODS

We partnered with a large, urban Federally Qualified Health Center (FQHC) with 320 providers across 44 primary care clinics located in Los Angeles and Orange Counties. These clinics have more than 1 million patient visits annually for predominantly Hispanic patients with high percentages preferring Spanish. In March 2018, the FQHC administered language proficiency assessments to evaluate whether providers and other patient-facing personnel were bilingual. They chose the Qualified Bilingual Staff (QBS) assessment to qualify support staff as bilingual with a passing threshold set at Level 2 which indicates “Capacity to offer services in the target language in a range of medical contexts” and the Clinician Cultural and Linguistic Assessment (CCLA) for providers requiring a minimum passing score of 80 out of 100. As part of a larger study on language concordance and interpreter use for Spanish-preferring patients, we interviewed providers about care delivery. We recruited providers who could provide insights and experiences about (1) language-concordant care (i.e., provider and patient are either Spanish-Spanish or English-English language concordant); (2) using professional, qualified interpreter services (via video or phone) (referred to hereafter as qualified interpreter services); or (3) using informal strategies for communication (i.e., not using the designated third-party interpreter services).

### Recruitment

**Figure 1:**
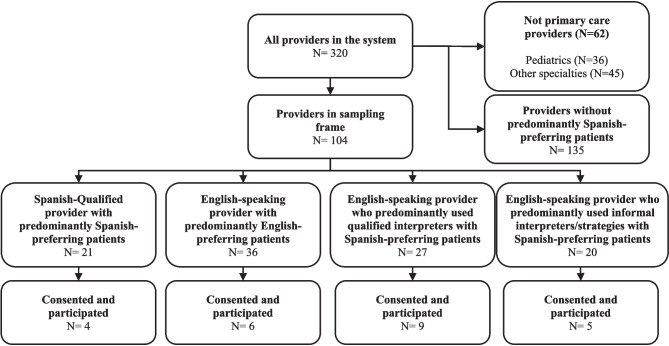
Sampling and recruiting flow diagram.

For 104 (of 320) primary care providers for adults, we received name; specialty; primary clinic location; years at the FQHC; Spanish-qualification status; number of face-to-face visits in the previous 6 months by English-preferring, Spanish-preferring, and other language-preferring patients; use of interpreter services in visits in the previous 6 months; sex; age; ethnicity; and contact information (Fig. 1). We excluded providers that predominantly cared for other language-preferring patients due to the heterogeneity of other languages represented. We stratified the remaining data and created sampling queues by two main characteristics: provider language fluency (Spanish-qualified or not) and provider type (i.e., provider’s predominant language scenario for in-person visits over the 6 months). Provider type included those who predominantly (1) provided patient-provider language-concordant care (either Spanish-Spanish or English-English); (2) used qualified interpreter services; and (3) used informal interpreter/communication). We aimed to recruit eight to nine providers of each type. We sent recruitment emails in batches of 10 to all providers in a sampling stratum and up to five weekly follow-up emails. We also called provider offices up to five times for providers for whom we had telephone numbers.

### Data Collection

We conducted 24 phone interviews with providers from May 30 to August 30, 2023. Providers gave verbal consent to participate and be recorded at the start of the interview. All interviews were conducted by a member of the research team; notes were taken during the interview to use to finalize the transcripts of the audio-recordings. Audio-recordings were destroyed after transcripts were finalized. We used an IRB-approved semi-structured interview guide that was pilot tested with another provider. All interview participants received $100 for participating.

### Coding

We entered transcripts into Dedoose, a qualitative data analysis and research software program.^32^ We established codes based on the semi-structured interview guide and developed a code structure using systematic, inductive procedures to generate insights from responses.^33,34^ Three researchers did initial coding of the same early transcripts to identify topics and refine the codebook. We employed interrater reliability to refine codes and descriptions. We obtained a pooled kappa coefficient of 0.85, indicating “very good” coder agreement.^35^ Researchers independently coded remaining transcripts. We used regular team meetings to reach consensus on topics, identify discrepancies, define codes, and refine concepts and themes.^36^

### Analysis

We conducted conventional content analysis.^37^ Researchers identified themes derived directly from transcripts. We compared provider experiences for similarities and differences by whether providers were language-concordant, used qualified interpreter services, or used informal interpretation or other means of communication with Spanish-preferring patients; we examined theme frequency and salience across groups.^38,39^ We also compared those interviewed to those not interviewed by provider type across several provider and visit characteristics (e.g., years at FCHC, in-person visits in the previous 6 months) and found no differences.

## RESULTS

### Provider and Patient Characteristics

We interviewed 24 providers across 12 clinics. Figure 1 shows the number of interviews with providers by language concordance and type of interpreter use. Table 1 shows providers characteristics overall and by provider type. Providers using qualified interpreters spent less time working at the FQHC, whereas those using informal interpreters spent the most time at their site. Those with language-concordant relationships spent the fewest hours in direct patient care. Spanish-qualified providers had passed a fluency exam and were designated as qualified in Spanish; some other providers could speak Spanish but were not Spanish-qualified. Most individuals learned Spanish through school or formal education or grew up speaking Spanish. Providers not qualified in but who could speak Spanish or some Spanish reported issues with Spanish.

**Table 1:** Provider Characteristics, Overall, and By Provider Type

	Overall *N*=24	Language-concordant providers^#^ *N*=10	Providers using qualified interpreters *N*=9	Providers using informal interpreters *N*=5
	***Mean (standard deviation)***			
Years at FQHC	4.36 (3.96)	4.58 (3.22)	2.8 (2.64)	6.75 (6.33)
Years at clinic	3.12 (3.04)	2.65 (2.3)	2.57 (2.19)	5.05 (5.08)
Hours of direct patient care (per day)	7.22 (2.37)	6.42 (2.9)	8.11 (2.1)	7.2 (1.1)
Hours of direct patient care (per week)	34.33 (10.5)	29.5 (13.18)	38.78 (7.3)	36 (5.48)
	***N (percent)***			
Spanish qualified^&^ obtained via FQHC testing				
Yes	4^x^ (16.7%)	4^x^ (40%)	0 (0%)	0 (0%)
No	20 (83.3%)	6 (60%)	9 (100%)	5 (100%)
Speak Spanish obtained via interview				
Yes	7^x^ (29.2%)	4^x^ (40%)	0 (0%)	2 (40%)
Some^^^	5 (20.8%)	1 (10%)	3 (33.3%)	2 (40%)
No	12 (50%)	5 (50%)	6 (66.7%)	1 (20%)
Those who Speak Spanish or some Spanish were asked the following:	***N*=12**	***N*=5**	***N*=3**	***N*=4**
How learned Spanish *^+^				
Grew up speaking Spanish	4 (33.3%)	3^x^ (60%)	0 (0%)	1 (25%)
In school and/or formal education (i.e. in high school, college, medical school, Spanish classes)	7 (58.3%)	3^x^ (60%)	2 (66.7%)	2 (50%)
Lived in Spanish-speaking country	1 (8.3%)	0 (0%)	0 (0%)	1 (25%)
Worked/working with Spanish-speaking populations	3 (25%)	1 (20%)	1 (33.3%)	1 (25%)
Personal education (i.e., apps)	1 (8.3%)	0 (0%)	1 (33.3%)	0 (0%)
Self-reported Spanish fluency*				
Bilingual/fluent	6^x^ (50.0%)	3^x^ (60%)	0 (0%)	3 (75%)
Native speaker	1^x^ (8.3%)	1^x^ (20%)	0 (0%)	0 (0%)
Conversational^^^	5 (41.7%)	1 (20%)	3 (100%)	1 (25%)
Self-reported difficulty in Spanish*^+^				
None	5 (41.7%)	3^x^ (60%)	0 (0%)	0 (0%)
Medical terms	4 (33.3%)	1 (20%)	1 (33.3%)	2 (40%)
Writing	3 (25%)	1^x^ (20%)	2 (66.7%)	2 (40%)
Accents	1 (8.3%)	1 (20%)	0 (0%)	0 (0%)
Speed of speaking	3 (25%)	1 (20%)	1 (33.3%)	2 (40%)

^&^The Federally Qualified Health Center chose the Qualified Bilingual Staff (*QBS*) assessment to qualify support staff as bilingual with a passing threshold set at Level 2 which indicates “Capacity to offer services in the target language in a range of medical contexts” and the Clinician Cultural and Linguistic Assessment (*CCLA*) for providers requiring a minimum passing score of 80 out of 100, designating the providers as Spanish-qualified

^**#**^Includes Spanish-qualified providers who predominantly care for Spanish-preferring patients (*n*=6) and non-Spanish qualified, English-speaking providers who predominantly care for English patients (*n*=4)

^x^The count includes Spanish-qualified providers

^*^The denominator is those who responded “Yes” or “Some” when asked if they can speak Spanish

^+^Items that are not mutually exclusive, so percentages will add up to greater than 100% in each column

^^^The providers who reported speaking some Spanish

Table 2 summarizes clinic- and visit-level characteristics. Provider panels largely matched patient language preferences. About two-thirds of patients were Spanish-preferring. Providers who used informal interpreters had a slightly smaller proportion of Spanish-preferring patients. Nearly all providers reported at least 80% of staff at their clinic could speak Spanish and daily covered patients from other provider panels.

**Table 2:** Patient, Clinic, and Provider Characteristics, Overall, and By Provider Type

Provider-reported characteristics	Overall *N*=24	Language-concordant providers *N*=10	Providers that use qualified interpreters *N*=9	Providers that use informal interpreters *N*=5
	*Mean (standard deviation)*			
Percent of provider’s patient population’s language preference (per provider)*				
Spanish	66.21 (23.4)	67.9 (28.77)	70 (21.07)	56 (15.17)
English	32.54 (22.44)	29.6 (26.59)	30 (21.07)	43 (15.65)
Other language (e.g., Tagalog, ASL)	1.71 (5.07)	2.7 (7.85)	0.67 (0.5)	1.6 (1.95)
Percent of provider’s clinic’s patient population’s language preference (per provider’s clinic)*				
Spanish	68.29 (23.15)	67.9 (28.77)	76.67 (16.2)	54 (16.73)
English	30.46 (22.05)	29.6 (26.59)	23.33 (16.2)	45 (17.32)
Other language (e.g., Tagalog, ASL)	1.71 (5.07)	2.7 (7.85)	0.67 (0.5)	1.6 (1.95)
	*N (Percent)*			
Percent of provider’s clinic’s staff who speak Spanish (per provider’s clinic)*				
All/most	16 (66.7%)	8 (80%)	5 (55.6%)	3 (60%)
About 80%	7 (29.2%)	2 (20%)	3 (33.3%)	2 (40%)
One/a few	1 (4.2%)	0 (0%)	1 (11.1%)	0 (0%)
How often provider uses qualified interpreter services (per provider)*				
Almost all of the time	4 (16.7%)	0 (0%)	4 (44.4%)	0 (0%)
Most of the time	3 (12.5%)	0 (0%)	3 (33.3%)	0 (0%)
Some of the time	6 (25%)	2 (20%)	2 (22.2%)	2 (40%)
Rarely/never	11 (45.8%)	8 (80%)	0 (0%)	3 (60%)
How often provider covers for other providers (per provider)*				
Daily	19 (79.2%)	9 (90%)	7 (77.8%)	3 (60%)
Weekly	1 (4.2%)	0 (0%)	1 (11.1%)	0 (0%)
Sporadically	3 (12.5%)	1 (10%)	1 (11.1%)	1 (20%)
Never	1 (4.2%)	0 (0%)	0 (0%)	1 (20%)

^*^Items may add up to >100% due to rounding

We identified four main themes in our interviews.

### Theme No. 1: Providers Universally Noted That Speaking the Same Language as a Patient Is Preferable, But Not Feasible

All providers reported that language concordance ensured no loss of meaning or nuance in provider-patient discussions and interactions. With language concordance, providers did not need to think about how to adapt their thoughts for interpretation. As one Spanish-speaking provider noted:

[There are] a lot of benefits to speaking the same language as the patient. It is an easier flow with your words and with the patient. You are not thinking about whether you are using the right word or words.

Providers noted that speaking the same language leads to some differences in patient demeanor and openness. Another Spanish-speaking provider described it this way:

[When speaking the same language] I can see a physical relief in the patient, a relaxing in their body and a gratitude. Just like anything, there are sometimes where I think, “how do you say that? Did I explain it right?” but even through that, we can always work it out.

Providers noted that speaking the same language improves not just the visit, but post-visit care and follow-up. Another Spanish-speaking provider explained:

[Patients] want to continue to see you. Your follow up rate is higher, and no-shows are lower. [When you speak the same language] they will follow up with you. It seems like they are more willing to trust people when you speak the same language.

Most all providers noted that it is not always feasible for providers and patients to speak the same language. We heard from several providers that they appear to be the first provider that their patients have had who speaks fluent Spanish.

### Theme No. 2: Both Language-Concordant Providers and Those Using Interpreters Required Additional Time to Incorporate Strategies for Communication

All providers reported that caring for patients speaking a language other than English takes more time. One reason for this is that providers integrated additional strategies into the visit to bridge the communication gap. The most common strategies to support communication with Spanish-preferring patients included writing down instructions/explanations and, for providers not fluent in Spanish, using an interpreter. Table 3 provides exemplar quotes for the types of support provided to Spanish-preferring patients.

**Table 3:** Illustrative Quotes for Support Provided by All Providers to Spanish-Preferring Patients

Themes	Illustrative quotes	Interviewee role, provider type
*Support provided*		
Wrote instructions/explanations in Spanish	When I want to give patient instructions, I will write it in Spanish and have my nurse check it to make sure it is correct. Other times for labs, if we want to send notifications, if you are not speaking the primary language, then you want to provide additional detail. I use google translate and have some phrases that I have had my nurse draft with me. When you need to convey information and the interpreter is not used in those incidences, so in written communication.	*Physician, English speaking with predominant use of interpreter services*
Repeated information, provided after visit summary	I repeat what I said in the summary at the end… You get a sense with patients if they get it or not, especially if you know them for a while. The information is in the after-visit summary as well.	*Physician, English speaking with predominant use of interpreter services*
Used non-verbal forms of communication and visual aids	Pointing to body parts, talking about similar colors for descriptions. A lot of nodding, shaking my head no. Way more movements and non-verbal communication.	*Physician, self-reported Spanish speaking*
Relied on Spanish-written materials for patient education	For written materials, through the EMR, there is a whole library of educational materials and handouts in different languages. If I can find a handout in the patient’s preferred language, I include it in the after-visit summary or print out.	*Physician, self-reported Spanish speaking*
Approached shared decision making similarly	It is the same [for Spanish-preferring patients]. I discuss the options, the risks, and contra-indications. Sometimes I will intervene saying this is the best option based on your other medical conditions. I explain my choices and why one might be better. If there are no contraindications, they have the option to choose.	*Nurse practitioner, English speaking with predominant use of alternative approaches to interpreter services*
When using an interpreter, felt interpreter use made patients more likely to come back	[Not being able to speak Spanish] does not impact the panel. My patients like me. They tell the front they want to see me. Even if I don’t speak their language, they want to see me… I think it is my compassion. I have so much compassion… I tend to spend more time and make them understand I am there for them. Because of that, I am able to build trust and communication with them. A good rapport.	*Nurse practitioner, English speaking with predominant use of interpreter services*
When using staff or family members to interpret, did this to provide support for dialect, medical terms, health literacy, or cultural sensitivity	Maybe 10% have family that want to be the interpreter. That is patient preference. I feel that if I use the family only, they might not be able to express themselves using medical terms. It is almost always my preference to use the interpreter. I want my patients to understand my instructions and be comfortable.	*Nurse practitioner, English speaking with predominant use of interpreter services*

Providers varied in the amount and type of information they write down for Spanish-preferring patients, from short summaries of their care plan (i.e., listing new medications and their frequencies) to the whole care plan in Spanish (done by Spanish-qualified providers). For more complex cases, providers left a note for nursing or back-office staff to review the written instructions and explanations with patients to make sure the instructions were clearly understood.

Other strategies of language support included providers repeating information, sharing end of visit summaries, and using pictures/visuals or other non-verbal communication. Spanish-qualified providers used these other strategies least often. English language-concordant providers used some Spanish in their interactions with their patients who spoke Spanish (or another language), primarily to say hello and goodbye. Providers of Spanish-preferring patients often employed the teach-back method to confirm Spanish-preferring patients understood what was communicated.

Specific non-verbal communication strategies included pointing to important information to highlight and explain, including information in the patient medical record, on images and diagrams, and on medication bottles. Providers who did not speak Spanish would often add short Spanish phrases for extra emphasis. In some cases, providers would print visual aids such as patient education materials or anatomical diagrams to support communication and patient understanding.

Providers reported that educational materials in the FQHC medical record are available in both English and Spanish, specifically to support education for Spanish-preferring patients. Providers who cared for Spanish-preferring patients reported ensuring patients had post-visit education materials in their preferred language.

Providers reported that when patients did not want an interpreter or when other strategies for communication did not work that they would use staff or family members as informal interpreters. Patients might refuse to use a qualified interpreter because of past poor interpreter experience or because of a preference for a family member or friend to interpret. Some providers also reported using informal interpreters who shared a similar cultural background or Spanish dialect with the patient. Providers indicated that some patients needed information at a lower literacy level than in the written materials.

Providers using qualified interpreters reported that Spanish-preferring patients were likely to stay with them once they had an established relationship, even though interpreter use took more time. Providers who used informal interpretation and communication strategies reported similar experiences. Providers reported that there were some additional benefits to using family as interpreters because family and patients shared a common dialect and an understanding of health literacy and cultural background that is not typically shared with qualified interpreters.

### Theme No. 3: Using Any Interpreter Slowed Down Communication and Made a Visit Less Smooth. Using Qualified Interpreters Required Additional Time for Initiating Contact, Connectivity Issues and Conducting Consecutive Interpretation. Providers Reported That Interpreters Improved Communication Most But Not All of the Time

Providers using interpreters reported that the tradeoff in having better communication and patient experience was that communication was slower. Providers using a qualified interpreter (by video or phone) reported that the visit took more time primarily because of the need to have the interpreter listen to and repeat back in Spanish what was said by the provider and by the patient. Using a qualified interpreter also takes more time to initiate and set up the interpreter, to find an iPad to start interpretation, and for disruptions caused by poor Wi-Fi connections. Providers estimated that setting up an interpreter takes 3 to 5 min (of 15–20 min scheduled). Table 4 provides illustrative quotes from providers using qualified interpreters.

**Table 4:** Illustrative Quotes For Providers Using Qualified Interpreters

Identified themes	Illustrative quotes	Interviewee role, provider type
*Adds time to visit*		
Time needed to initiate interpreter (phone, video)	The interpreter would be ready when I go into the room in an ideal world. It takes a few minutes to get the information put into the system to use the interpreter, the patient ID, where we are calling from, and my ID. If that was improved and the interpreter is waiting, we don’t have to spend the 3–5 minutes getting things started.	*Physician (DO),* *English speaking with predominant use of interpreter services*
Time to find the iPad	We don’t have enough iPads. We have 17 exam rooms. There are only 8 of the iPads around. If they are moved, I have to find them, so that might be a delay.	*Physician, English speaking with predominant use of interpreter services*
Back and forth with provider-interpreter-patient	I see the major difference with Spanish-speaking patients is there are more pauses because of the translator. I cannot go on for a paragraph. They might be able to keep up and translate correctly. I do 1–2 sentences at a time and then keep going. It takes longer because of that.	*Physician (DO), English speaking with predominant use of interpreter services*
Needed to speak one sentence at a time	There is variation in interpreter quality. Some cannot have me speak more than 1 sentence at a time. My narrative is different. For English, I can tell a story, give examples, explain why I am doing things. For Spanish, I have to go back and forth. There are less opportunities to explain things in more detail or provide examples.	*Physician, English speaking with predominant use of interpreter services*
Needed to explain medical terms	Interpreters google medicines or something I say. I can see they are looking something up and there is a delay. Sometimes they will botch it. They will say it in Spanish, and I can tell. Sometimes they will repeat what I said and just mumble it to the patient.	*Physician, English speaking with predominant use of interpreter services*
Technical issues (e.g., Wi-Fi, poor sound quality, freezing)	Sometimes the connection is not great. We are in the middle of the conversation, and it cuts off. Then I have to click the screen again to re-connect. I think the biggest challenge is connectivity. Sometimes the interpreter cannot hear me, so I have to speak up, or the connection is bad. The screen will freeze.	*Nurse practitioner, English speaking with predominant use of alternative approaches to interpreter services* *Nurse practitioner, English speaking with predominant English-preferring patients*
*Improves communication*		
Some low-quality interpreters	The translators are not all the same. Sometimes, I might pick up on words and sentences. I can figure out if the translator is interpreting exactly what I am saying. They might not interpret it correctly. I will say this is what I meant; can you explain it this way? This helps the patient understand where I am coming from. If I don’t pick those up, that may be a problem.	*Physician (DO), English speaking with predominant use of interpreter services*
Misunderstandings, miscommunication	They can express what they want me to know, their feelings, and what they want me to know. If I speak in English, they only use a few words. If I use the translator, they will speak more. When I use the translator, I can explain everything.	*Nurse practitioner, English speaking with predominant use of alternative approaches to interpreter services*

Providers stated that the use of qualified interpreters most often improved patient experience, particularly patient-provider communication. With qualified interpreters, providers reported they felt their patients understood what they were saying and that patients could communicate their concerns. Providers also reported that they felt their patients appreciated interpreter services, adding some patients did not have access to interpreters with providers outside of the study’s FQHC. Most providers reported no impact of using an interpreter on continuity of care or missed appointments.

Providers who do not speak Spanish would often cut patient education from discussion during a visit, as education was time-intensive, and patients prioritized other concerns. Because bilingual written materials exist (and are legally required by Section 1557),^1^ providers reported feeling comfortable briefly reviewing the materials with their patients (and with an interpreter if necessary) and asking the patient to review the material after the visit. Providers also reported asking nursing or back-office staff to review key pieces from the educational materials with the patient.

Although these communication and comprehension strategies took additional time, providers said their approach to shared decision making with Spanish-preferring patients did not change, even if this required going over the regular visit time. Some providers said they would volunteer their recommendation and justification to patients to save time, but still made sure to present the available options and related information on treatments for patients to select.

Providers that typically chose to use informal interpreters or other communication strategies reported more past difficulties, such as poor audio or video quality, with formal interpretation services. Some cases providers reported having a low-quality qualified interpreter, which required more following up with the interpreter during the visit to clarify or correct what they stated or calling for a new interpreter. To compensate for low-quality interpreters, providers noted that they would speak one sentence at a time, explain medical terms to the interpreter, or have a nurse follow-up if it was clear the interpreter did not understand the medical content or terminology.

### Theme No. 4: Provider Experiences —Including Approaches to Care and Communication Support— Qualitatively Differed by the Language of the Provider and Their Use of Interpreters

Providers’ experiences of providing care to Spanish-preferring patients qualitatively differed by the provider’s language and use of interpreters. Visits differed by types of communication support, concerns elicited, topics covered, patient comprehension, the range of sensitive topics discussed, and time spent on rapport-building and patient education. Table 5 shows illustrative quotes on these themes.

**Table 5:** Illustrative Quotes For Providers’ Qualitatively Different Experiences

Identified themes	Illustrative quotes	Interviewee role, provider type
*S*panish-Spanish concordant experiences		
Patients shared more issues due to comfort level	Patients tell me a million more things because I speak Spanish. It takes longer because the patient says I really want to tell someone this because I never had time, or I couldn’t because they couldn’t understand me. I have to redirect and say I want to help but let’s focus on the 5 and then make another appointment. It is because I speak Spanish. They fit as much as they can because we can go faster.	*Physician, Spanish speaking*
Needed translation for dialect or specific terms in Spanish	I have used it before for mental health. I have used it before when discussion radiology reports. CT scans, MRIs. There is terminology I don’t know. I do not want that information to be misinterpreted especially if it is an unfortunate result. Lastly, when it is a different dialect, Portuguese or Spanish.	*Physician assistant, Spanish speaking*
No issues eliciting concerns from patients	I try to standardize the start and end of a visit. It makes sure I provide instructions, review medical history, and making it the same for every visit.	*Physician, Spanish speaking*
No issues discussing sensitive topics with patients	For STDs, I would ask for the same thing, to use the tools to help for educating. Then, I can get my message across to the patient. I don’t feel any different when discussing with the patient, speaking Spanish with the patient. I have the same type of education regardless.	*Nurse practitioner, self-reported Spanish speaking*
*Discordant experiences using interpreters*		
Less smooth, stilted flow of information exchange	I see the major difference with Spanish-speaking patients is there are more pauses because of the translator. I cannot go on for a paragraph. They might be able to keep up and translate correctly. I do 1–2 sentences at a time and then keep going. It takes longer because of that.	*Physician (DO), English speaking with predominant use of interpreter services*
More short and directed statements	I use the interpreter and then print out the handout to have them better understand it. We have to use short sentences for the interpreter and the patient to ensure understanding. I want to make sure they understand what I mean and what they need to do.	*Nurse practitioner, English speaking with predominant use of interpreter services*
Less time on building rapport	Some of my English-speaking patients I have a good rapport with… It is easier to build a relationship because you can speak the same language.	*Nurse practitioner, English speaking with predominant use of interpreter services*
Asked patients to repeat back to ensure understanding	I make them repeat things [to make sure they understand]. Before I end the conversation, I ask if they have any other questions.	*Nurse practitioner, English speaking with predominant use of interpreter services*
Often used follow-up visits to address concerns	All providers have to get out on time, want to make sure we are meeting patient needs. We hit the necessary pieces like take this medication and we are increasing the dose and maybe will cover the diet and exercise next time.	*Physician (DO), English speaking with predominant use of interpreter services*
*Discordant experiences not using interpreters and do not speak Spanish fluently*		
Asked more discrete questions	With patients who do not speak the same language, I ask more discrete questions about the concerns. I don’t think it is too different. I do the same thing with those patients.	*Physician, self-reported Spanish speaking*
Asked more if patient understood what was said	If I sense there is some confusion, then I will double check to make sure they understand what is being said. Asking if they comprehend and if not, trying to repeat it.	*Physician, self-reported Spanish speaking*
Relied more on staff to ensure patient comprehension	When you deal with a patient who speaks Spanish or another language, you have to take more time. It will be more difficult to communicate. At the end of the visit, we always make sure they understand what you said. You have the back office there to check with the patient to make sure the patient understands what is going on.	*Nurse practitioner, English speaking with predominant English-preferring patients*
Needing caregivers	For elderly patients and chronic conditions, I talk more to the caregiver. If the caregiver does not understand, they don’t know how to help with the medications. The only difference is or the caregiver in Spanish, you need to spend more time with them. You have to use simple words and have to give time to check if they understand. You have to be patient and repeat until they understand.	*Nurse practitioner, English speaking with predominant use of alternative approaches to interpreter services*
End of life care	For Advanced Directives, it is something I want to have a family member around for. I want to bring the family in to be involved. They can join by the phone as well, but I prefer they be there.	*Nurse practitioner, self-reported Spanish speaking*

### Spanish-Spanish Concordant Providers

Spanish-qualified providers reported taking more time with Spanish-preferring patients than with English-speaking patients for two reasons. First, Spanish-preferring patients felt comfortable sharing more issues with them. Second, regional or dialect differences could require even Spanish-qualified providers to use an interpreter or look up what the patient was saying. Nevertheless, these providers reported no differences in eliciting concerns with their Spanish-preferring patients than with their English-preferring patients.

### English-Speaking Providers Using Interpreters

English-speaking providers who predominantly cared for Spanish-preferring patients reported that visits with Spanish-preferring patients “run less smoothly.” Conversational flow was interrupted by the need to use an interpreter, having to break up explanations and instructions into discrete chunks, delays in questions and responses going through an interpreter, and sometimes by low-quality interpretation and the need to switch interpreters. These providers often shortened their sentences to facilitate communication through the interpreter. Providers using interpreters reported providing more detailed (often written) information to their patients on complicated issues and asking if patients had any more questions. Providers using qualified interpreters reported little discussion on sensitive topics such as mental health or sexual/reproductive health, but this may have been a result of having less time with patients.

Providers using qualified interpreters used a wider variety of approaches to improve patient comprehension. This included engaging nurses or staff to clarify information for patients and asking patients to repeat back information. Providers with language discordance mentioned more often wanting/needing a qualified interpreter to review and care for patient’s mental health, convey nuance, allow for higher levels of cultural sensitivity, and discuss chronic conditions.

Providers using interpreters (qualified or informal) reported adjusting their communication approach, spending less time building rapport, and focusing on short, directed statements to address as many patient concerns as possible. When there was too much to discuss in a given visit, these providers reported using follow-up visits to address remaining concerns.

Providers who were not fluent in Spanish and who did not use an interpreter also employed a wider range of linguistic support activities and reported more often asking their patients if they understood what was being communicated. English-speaking providers would engage a nurse or staff member to clarify information (in Spanish) and ask the patient to repeat information to confirm understanding. Providers using informal interpreters or other methods of communication reported a wider range of sensitive topics, highlighting a broader set of topics that may need language support.

## DISCUSSION

We found that providers preferred to speak the same language as the patient, employed varying language support strategies, and required more time to care for Spanish-preferring patients for various reasons, and that using interpreters did not always improve communication. Also, providers may not be aware of the patient’s legal right to an interpreter and the standards around language access resources that are outlined in Section 1557.

Time pressure during primary care office visits, especially for providers with larger numbers of psychosocially complex and limited English proficient patients, is well documented.^40–45^ Our findings identified several barriers that organizations need to address related to time pressures within a given patient visit for Spanish-preferring patients. Providers reported consistently that caring for Spanish-preferring patients required additional time to incorporate strategies for communication. These strategies primarily included writing out instructions and explanations for patients in Spanish and spending more time repeating information, reviewing after-visit summaries, utilizing non-verbal forms of communication, and using pictures/visuals. This suggests that organizations need to account for more time during face-to-face visits with patients who do not speak English (i.e., in our study prefer Spanish).

Previous research has also identified several barriers to providing care for patients with limited English proficiency.^46,47^ Providers in our study reported that using *any* interpreter prolonged visits, slowed down communication, and made the flow of the visit more stilted, and that using *qualified* interpreters took even more time because of the need to setup and initiate the use of an interpreter via phone or iPad. The interpretation techniques used (e.g., shortening sentences, clarifying understanding across three people) required providers to focus on one or a few main issues and there were concerns raised about not addressing all of the patients concerns. Often providers scheduled follow-up appointments to address these other patient concerns; some chose to prolong the visit time to address patient concerns. Providers said interpreters improved communication most but not all the time, noting interpreters’ difficulty with medical content and terminology, requiring provider clarification or staff to further translate. This suggests that organizations need to systematically track and review the concerns and care addressed at each visit and across visits for Spanish-preferring patients who are regularly treated by a provider using an interpreter, including scheduling follow-up care for any concerns not addressed. Concerns raised about the quality of interpretation suggest that organizations need to proactively collect provider input on the quality of specific interpreters, review the type and background of interpreters offered, and regularly audit the quality of interpretation.

Our findings laid out several differences across visits by language spoken and use of an interpreter that add to previous research on language concordance and interpretation in primary care. Language-concordant providers and those requiring an interpreter reported that they approached shared decision making with Spanish-preferring patients similarly. Nevertheless, they noted differences in eliciting concerns, discussing sensitive topics, what is covered within a visit, patient comprehension, and time spent on rapport-building and patient education.

It is troubling that despite numerous studies describing time pressures for clinicians caring for Spanish-`preferring patients and language issues related to interpretation that successful and effective strategies have not been identified or evaluated. Our findings support the need for research that identifies and tests communication support strategies for providers and for clinics to employ when caring for Spanish-preferring patients. Given the differences in provider experiences and patient visits reported in our study, organizations may want to undertake quality improvement efforts to better understand the experiences and barriers present in face-to-face visits. Reviewing the workflow (i.e., from waiting areas to exam rooms to after visit processes) and efficiency of activities that may require interpretation need to be conducted. This could be done using shadowing or peer-observations techniques to document effective provider strategies, workflow improvements, and needed patient education materials or decision aids to support effective provider-patient communication.

Our study has several limitations. We studied one organization’s experience with language-concordant providers and the use of interpreters (the two main primary care strategies engaged in nationally), limiting the generalizability of our findings. Utilizing interpreters and language-concordant providers in health care organizations serving less vulnerable populations may involve different challenges. We also were not able to interview all providers with predominantly Spanish-preferring patients. As such we may have only included those with strong feelings toward using interpreters and supporting patient communication needs. Nevertheless, our sample was purposively sampled to pull individuals from strata by provider type and patient visit type, so a variety of provider perspectives were included. The results of non-response bias analysis indicated no difference across those interviewed/not interviewed. We also were not able to include the voice and perspectives of interpreters. Future research is needed that explores the use of interpreters in primary care and their effect on and health care outcomes, such as continuity of care, medication adherence, and receipt of preventative care. A broader, multi-site evaluation may identify additional insights, lessons learned, and challenges.

Providers caring for Spanish-preferring patients employ a large variety of different language supports and communication techniques that vary by language concordance or interpreter use. Providers described several barriers that organizations need to address related to time pressures and language interpretation issues within a given patient visit for Spanish-preferring patients. Research is needed that identifies and tests language support strategies for providers and clinics to employ when caring for Spanish-preferring patients, as well as on structural changes within clinics to preserve time during patient visits for more discussion of health care needs.

## Data Availability

Data sharing is not applicable for this article as confidentiality was given the interviewees who participated in the study.
